# Pilot Study of the Effects of Bright Ambient Therapy on Dementia Symptoms and Cognitive Function

**DOI:** 10.3389/fpsyg.2021.782160

**Published:** 2021-12-24

**Authors:** Chuen-Ru Liu, Yiing Mei Liou, Jwo-Huei Jou

**Affiliations:** ^1^College of Nursing, National Yang Ming Chiao Tung University, Taipei, Taiwan; ^2^Psychiatric Nurse of City Hospital, Songde Branch, Taipei, Taiwan; ^3^Department of Materials Science and Engineering, National Tsing Hua University, Hsinchu, Taiwan

**Keywords:** light therapy, dementia, behavioral and psychological symptoms of dementia, cognitive function, bright light therapy

## Abstract

Light therapy potentially improves dementia symptoms. In this study, we examined the effects of bright light therapy on neuropsychiatric behaviors and cognitive function. Thirty-five participants were assigned to either the experimental or comparison group. The experimental group was exposed to bright light at 2,500 lux, and the comparison group was exposed to 114–307 lux. The instruments used were the Neuropsychiatric Inventory and the Mini-Mental State Examination. The experimental group showed a significant improvement in Neuropsychiatric Inventory scores; these scores, which were calculated using generalized estimating equations with medication (benzodiazepines) as a covariate, were reduced by 65% *(P* < 0.001) and 78% (*P* = 0.001) by the 5th and 9th weeks, respectively. At the same time, Mini-Mental State Examination scores increased by 19% (*P* = 0.007) and 28% (*P* = 0.04), respectively. However, differences in outcomes between the 5th and 9th weeks were not significant. A 4-week regimen of bright light therapy was the most effective, with higher adherence and acceptability.

## Introduction

Approximately 55 million people have dementia worldwide, and this number is expected to increase to 78 million by 2030 and 139 million by 2050 (World Health Organization, 2021). Thus, dementia-related health issues have become a high-priority topic.

Behavioral and psychological symptoms of dementia (BPSD) are nearly universal in patients with dementia (Lyketsos, 2007). BPSD are the predominant factors that increase the burden of caregivers of older adults with dementia (Melo et al., 2011). BPSD comprise a group of heterogeneous symptoms induced by dementia, the most frequent of which are depression (42%), aggression (40%), anxiety (39%), and sleep disturbances (39%) (Zhao et al., 2016). BPSD may be improved with pharmacological treatment; however, these therapeutics may expose patients to various adverse side effects, including sedation, dysphagia, falls, delirium, and worsening cognitive symptoms (de Oliveira et al., 2015). Non-pharmacological alternatives for BPSD management include light therapy, cognitive rehabilitation, and Snoezelen multisensory stimulation therapy, but studies of these treatments have reached inconsistent conclusions (de Oliveira et al., 2015; Duan et al., 2018; Law and Kwok, 2019).

Light therapy is a relatively harmless and cost-effective intervention method, and artificial light exposure has many advantages over natural light; for example, artificial light therapy is not influenced by weather and has no adverse effects on the skin. The benefit of light stimulation decreases as the cornea ages and as mobility decreases, and thus people with dementia are unable to expose themselves to sunlight and regulate their circadian rhythms (Lyketsos, 2007). During the aging process, bright light therapy may activate the residual activity of their presumably weakened circadian system (Rubiño et al., 2020). Thus, a proper regimen of artificial light has become crucial for people with dementia.

Light therapy is effective at improving cognitive function (Yamadera et al., 2000; Riemersma-van der Lek et al., 2008), depressive symptoms (Riemersma-van der Lek et al., 2008; Figueiro et al., 2014; Onega et al., 2016), agitation/aggression (Yamadera et al., 2000; Burns et al., 2009; Figueiro et al., 2014; Onega et al., 2016; Law and Kwok, 2019), BPSD (Skjerve et al., 2004; Dowling et al., 2007; Riemersma-van der Lek et al., 2008), and sleep disturbances (Yamadera et al., 2000; Skjerve et al., 2004; Dowling et al., 2005; McCurry et al., 2005, 2011; Sloane et al., 2007; Riemersma-van der Lek et al., 2008; Figueiro et al., 2014). The effects of light on circadian rhythms, sleep, and mood are mediated by intrinsically photosensitive retinal ganglion cells (Fernandez et al., 2018; Yanar and Halassa, 2018). These effects of light mediated by intrinsically photosensitive retinal ganglion cells involve pacemaker-independent input to the suprachiasmatic nuclei (Fernandez et al., 2018; Yanar and Halassa, 2018). Light exposure suppresses melatonin secretion and induces serotonin secretion, which mitigates geriatric depressive and anxious moods and improves attentiveness. Additionally, daylight induces the internal biological clock to synchronize with the changing external environment (synchronization of circadian rhythms), reducing phase delay and night awakening and thus reducing sleep disturbances (Shikder et al., 2012; Forbes et al., 2014; Missotten et al., 2019; Wirz-Justice and Benedetti, 2020). Furthermore, the risk of strange behavior and psychological symptoms is reduced (Fernandez et al., 2018), and cognition is enhanced (Yamadera et al., 2000; Riemersma-van der Lek et al., 2008; Forbes et al., 2014; Rubiño et al., 2020).

However, meta-analyses of light therapy have been somewhat inconsistent, and the heterogeneous effects of light treatment on dementia are not yet fully explained (Forbes et al., 2009, 2014; Shikder et al., 2012; Hanford and Figueiro, 2013; van Maanen et al., 2016; Chiu et al., 2017; Mitolo et al., 2018; Missotten et al., 2019). Some of the factors influencing its effectiveness are light intensity, spectral composition, duration, timing, exposure patterns, dementia type, sleep patterns, dementia severity, and acceptance of and adherence to the regimen (Forbes et al., 2009, 2014; Shikder et al., 2012; Hanford and Figueiro, 2013; van Maanen et al., 2016; Chiu et al., 2017; Figueiro, 2017; Mitolo et al., 2018; Missotten et al., 2019; Faulkner et al., 2020).

In the present study, we used a lighting intervention model with horizontal illumination of at least 2,500 lux (McCurry et al., 2005, 2011; Dowling et al., 2007; Sloane et al., 2007; van Maanen et al., 2016; Chiu et al., 2017; Missotten et al., 2019) within a 45° visual field (McCurry et al., 2005, 2011) for at least 60 min/day in the morning from 9 am to 10 am (Dowling et al., 2005, 2007; Zhou et al., 2012; van Maanen et al., 2016; Faulkner et al., 2020) for at least 2–4 weeks (Skjerve et al., 2004; Figueiro et al., 2014; Figueiro, 2017; Missotten et al., 2019). The treatment was administered in a group-based setting to improve adherence and acceptability for older adults with dementia (McCurry et al., 2011; van der Wardt et al., 2017; Vseteckova et al., 2018). We sought to increase the compliance of older adults with dementia using this regimen of ambient light therapy (Hickman et al., 2007; Burns et al., 2009; Barrick et al., 2010). The proposed bright light therapy model could be easily implemented as a non-pharmacological intervention as part of practical care for older adults with dementia to explore its feasibility and potential benefits.

The purpose of this pilot study was to compare the effects of bright light therapy models and general lighting in the morning on the BPSD and cognitive function of older adults with dementia through longitudinal experiments.

## Materials and Methods

### Design

As a non-randomized controlled pilot study with a convenience sampling design, this study utilizes a single-blind approach. We recruited participants from communities and nursing homes in Taiwan between April and December 2019 through social networking tools, including Line and Facebook. Participants in the experimental and comparison groups were exposed to bright light therapy (2,500 lux) and general lighting, respectively.

### Participants

The calculated sample size was 20 participants using G*Power 3.1 computer software, and the data were analyzed using analysis of variance with the conditions set to a statistical significance of α = 0.05, β = 0.2 and an effect size of *f* = 0.39 (McCurry et al., 2005). *F* tests and repeated measures ANOVA were used to determine within-between interactions.

Participants were selected based on the following inclusion criteria: (1) diagnosis of dementia consistent with the Diagnostic and Statistical Manual of Mental Disorders, 5th edition (Kupfer et al., 2008); (2) age between 60 and 95 years; (3) consent to participate in the study obtained from the participants and their guardians; and (4) willingness to participate in the group. Participants were excluded if they (1) had adverse reactions to light, such as systemic lupus erythematosus, epilepsy, blindness, retinal detachment, and macular degeneration; (2) were on the sleep cycle of an advanced sleep-wake phase disorder; and (3) had a score < 3 points on the Mini-Mental State Examination (MMSE) and were unable to express their own intentions verbally.

**FIGURE 1:**
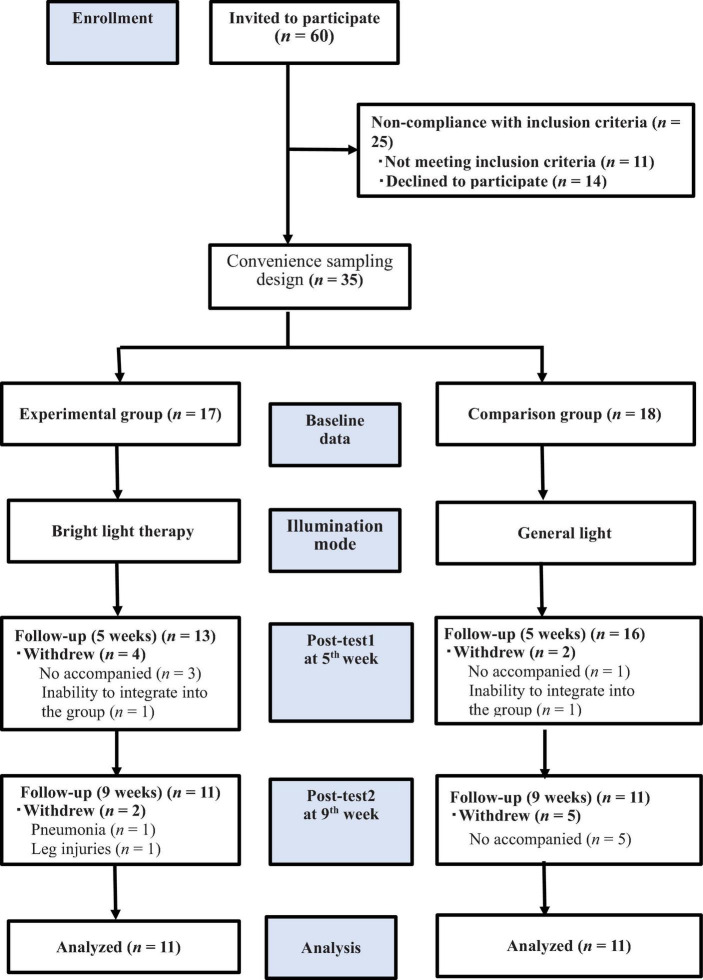
The flow chart of the study. We included 35 patients who underwent the baseline evaluation and postintervention assessments in the 5th and 9th weeks. allocated them to the experimental (*n* = 17) and comparison (*n* = 18) groups; only 22 patients completed the study, and the experimental and comparison groups were exposed to bright light therapy (2,500 lux) and general lighting, respectively.

Among the 60 individuals invited to participate in the study, 11 did not meet the inclusion criteria, and 14 declined to participate. Accordingly, we included 35 patients and consecutively allocated them to the experimental (*n* = 17) and comparison (*n* = 18) groups. Thirteen patients withdrew from the study for the following reasons: unable to attend on their own and unavailability of family members to accompany them to daily therapy (*n* = 9), pneumonia or leg injuries (*n* = 2), and inability to integrate into the group (*n* = 2). Finally, only 22 patients completed the study (Figure 1). Patients in the experimental group received treatment at the light center of National Yang Ming Chiao Tung University. In contrast, patients in the comparison group received light exposure at the institutionalization modalities center. The patients were transported to the experimental site using a bus equipped with special wheelchair equipment or were escorted by family members 5 days a week.

#### Experimental Group: Bright Light Therapy Model

**FIGURE 2:**
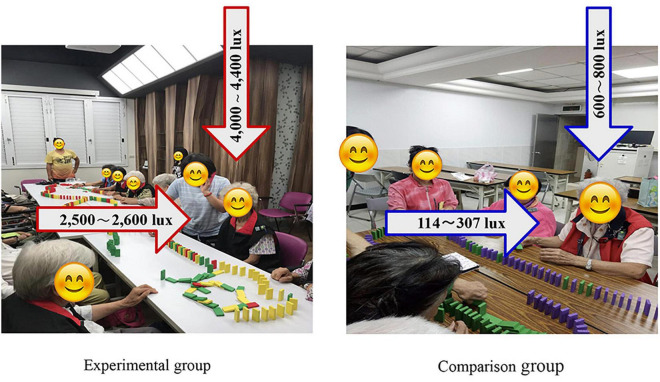
Design of ambient conditions for exposure to bright light therapy and general lighting.

Participants in the experimental group were exposed to bright light therapy evenly under ambient conditions of horizontal illumination of 2,500–2,600 lux and a vertical illumination of 4,000–4,400 lux from a panel light (full-spectrum light, products developed by the National Yang Ming Chiao Tung University industry-university cooperation) in a special room. The ambient lights were placed on the ceiling within a 45° visual field with at least 2,500 lux, full-spectrum light to provide exposure (Figure 2). Bright light therapy exposure occurred for at least 60 min/day from 9 am to 10 am, Monday through Friday, for 8 weeks, resulting in 40 h of exposure. Bright light therapy was coupled with group participation. The horizontal illumination started at 500 lux and increased by 500 lux daily until it reached 2,500 lux; the light was then maintained at 2,500 lux until the end of the intervention.

The participants were seated on a chair approximately 1.2 m away from the artificial ambient exposure source at eye level. We used the Konica Minolta CL-500A (illuminate monitors illuminance and color temperature) illuminometer to ensure that the bright light therapy was standardized at 2,500 lux using the same effective parameters in each session throughout the study period. The lights in the surrounding environment were switched off, the curtains were drawn to help limit artificial ambient exposure, and the special aluminum windows completely blocked the external light. We used a light measuring instrument to ensure the consistency of light exposure with the experimental parameters. We provided sunglasses for the participants to wear before going outdoors to reduce sunlight interference.

Group-based light therapy was introduced to promote the adherence of older adults with dementia to participation in bright light therapy. This approach was coupled with a daily group session that included reminiscence, art, music, art creation, DIY creation of essential Jawoongo oils, viewing photos, and sharing of family mottos and heirlooms, great past achievements, unfulfilled travel experience wishes, and my ideal funeral, for a total of 40 h of group.

A token was provided for each period of participation, which was exchangeable for gifts. Additionally, a bus with special wheelchair equipment was used to transport the participants home or to the institution. Next, they would have indoor rest and lunch and no longer be exposed to outdoor sunlight in the morning.

#### Comparison Group: General Light

The participants in the comparison group were exposed to general lighting with a horizontal illumination of 114–307 lux and a vertical illumination of 600–800 lux (Figure 2). The room used for the comparison group had no windows, and some of their rooms had curtains to maintain the same illumination in each session. The group activities were the same for both the experimental and comparison groups.

### Instruments and Outcome Measures

The measurement tools included demographic data and two outcome measures: BPSD were determined using the Neuropsychiatric Inventory (NPI), and cognitive function outcomes were obtained using the MMSE. The NPI data were obtained by interviewing the corresponding caregivers and nursing home staff, while the researchers directly obtained the MMSE data. The data were collected at baseline and in the 5th and 9th weeks from April to December 2019. The data collectors were single-blinded to the participants.

### Participant Demographic Data

The demographic data included sex, age, educational level, marital status, sleep pattern, dementia type, source, dementia severity, and medication use (benzodiazepines, antidepressants, antipsychotics, and anti-dementia drugs). They were obtained from the primary caregiver and nursing home staff.

### Sleep Pattern

According to the reports of caregivers and nursing home staff, sleep and wake times were observed for a week. Additionally, patterns were classified as advanced sleep-wake phase disorder, delayed sleep-wake phase disorder, irregular sleep-wake rhythm disorder, or a sleep-wake rhythm (Burgess and Emens, 2016), and accelerometer-monitored sleep pattern data were recorded using an accelerometer. Compared with sleep logs of older adults with dementia, the between-measurement error value was < 30 min. The accelerometer (XA-5, Taipei, Taiwan) was worn around the wrist to continuously record actigraphic data for ≥ 3 days at baseline using the KY laboratory software package^1^.

### Defined Daily Dose

The defined daily dose (DDD), which is promoted by the World Health Organization (Polk et al., 2007), is the assumed average maintenance dose per day for a drug used for medication in adults. The DDD represents a unit of measurement only and was calculated as the total dose of a drug divided by the DDD. The drug data were collected at baseline and in the 5th and 9th weeks to monitor the effect of drug changes on the study results.

### Neuropsychiatric Inventory

The NPI was developed by Cummings et al. (1994) (see also Connor et al., 2008; Lai, 2014) to assess dementia-related psychopathology and neuropsychiatric behaviors. The NPI includes four domains: emotional symptoms (e.g., dysphoria, anxiety, apathy, euphoria, and irritability), psychiatric symptoms (e.g., delusions and hallucinations), behavioral problems (e.g., agitation, disinhibition, aberrant motor behavior, appetite, and eating abnormalities), and sleep disturbances (e.g., nighttime behavioral disturbances) (Connor et al., 2008). The NPI shows good content validity, concurrent validity, and interrater reliability. The caregivers were asked to rate the frequency of the symptoms of each disturbance on a scale of 1 (occasionally or less than once a week) to 4 (very frequently, more than once a day, or continuously). The rating of the symptom severity was 1, 2, or 3 for mild, moderate, or severe, respectively. The total score ranged from 0 to 144 points, where a higher score reflects a more severe level of BPSD. The NPI subdomains were significantly correlated with the domains of behavioral disturbances and the Hamilton Rating Scale for Depression. Interrater reliability ranged from 93.6 to 100%, depending on the subdomain, and test-retest reliability was also high: *r*(20) = 0.79 (Cummings et al., 1994; Connor et al., 2008; Lai, 2014; Cloak and Al Khalili, 2021). In this study, the NPI exhibited adequate overall internal consistency (α = 0.66). Cronbach’s α coefficients for the older adults with dementia were 0.56 and 0.75 for the pretest and posttest, respectively.

### Mini-Mental State Examination

The MMSE was developed to quantitatively assess dementia-related cognitive function, ranging from 0 for the worst to 30 for the best (Folstein et al., 1975; Graf et al., 2001; Baek et al., 2016). It has good test-retest reliability (0.80–0.95), sensitivity, and specificity in detecting mild dementia. The MMSE is categorized into orientation [e.g., orientation questions, five each for time and place (10 points)], registration [e.g., three-word registration and 1-min recall (3 points)], attention and calculation [e.g., assessed either by serial subtraction of 7 from 100 or serial subtraction of 3 from 20 (5 points)], recall [e.g., three-item recall test for memory (3 points)], and language, visuospatial construction [assessed by a three-stage command, repetition, naming, reading comprehension, and writing (8 points) and copying two intersecting pentagons (1 point)] (Graf et al., 2001). In this study, the MMSE exhibited an adequate overall internal consistency (α = 0.85). Cronbach’s α coefficients for the older adults with dementia were 0.81 and 0.88 for the pretest and posttest, respectively.

### Dementia Severity

Mini-mental state examination scores ≥ 21 indicate the presence of mild dementia, 11–20 indicate moderate dementia, and 0–10 indicate severe dementia (Perneczky et al., 2006).

### Statistical Analyses

The demographic characteristics of the participants in the two groups were analyzed using SPSS version 24.0 (IBM Corp., Armonk, NY, United States), with sex, educational level, marital status, sleep pattern, dementia type, dementia severity, and source taken into consideration. These descriptive statistics were performed at baseline and in the 5th and 9th weeks to examine whether the demographic characteristics were affected by the loss of any of the participants. Descriptive statistics are presented as the number of cases (n), percentage (%), mean, and standard error for both the experimental and comparison groups. The primary pretest-posttest analyses were based on the intention-to-treat sample. Non-parametric inferential statistics (chi-square test and Mann–Whitney *U* test) were used to compare the basic profiles of the two groups before and after the intervention and within groups. The Mann–Whitney *U* test was used to assess within-group differences in age, NPI and MMSE scores, and medication (benzodiazepines, antidepressants, antipsychotics, and anti-dementia drugs) to compare the baseline values of the two groups. The data were collected at three time points: baseline and after light exposure interventions (in the 5th and 9th weeks). One unit of the drug was defined as the DDD. Considering the possible consequences of repeated measurements, the intervention effects were tested using generalized estimating equations for comparisons between groups (Zeger and Liang, 1986). Improvements in outcomes (NPI and MMSE scores) over time in both groups were analyzed using generalized estimating equations with an exchangeable working correlation matrix. A robust standard error was used to calculate statistical significance. We tested for the main effects of the groups (experimental and comparison) and time points (baseline, 5th week, and 9th week), as well as their interaction (group × time point); a statistically significant interaction effect (group × time point) indicated that the two groups exhibited significantly different changes over time. Benzodiazepine use was included as a covariate in the generalized estimating equations (Rosenberg et al., 2012).

### Feasibility: Retention, Attendance, and Adverse Events

All the participants from the experimental and comparison groups completed profile tracing. Thirty-five participants completed the baseline assessment, and 29 and 22 participants completed the 5th and 9th week assessments, respectively, for a retention rate of 64.7% in the experimental group and 61.1% in the comparison group. The attendance rate was 93% in the experimental group and 75% in the comparison group. No adverse effects were observed (e.g., fall, injury, eye injury, headache, and dizziness) during the 8 weeks of light therapy.

### Treatment Fidelity

The participants were recruited by a candidate psychiatric nurse who was confirmed by the doctoral supervisor to have met the academic rigor, was briefed on the research plan before implementation, and had received training to ensure the consistency of data collection. The principal researcher met with the first author to ensure that the protocols were being followed, establishing the interrater reliability of the evaluator. The intervention was delivered by the first author, who had completed training before program implementation. They intervened in accordance with standard procedures.

### Ethical Considerations

Participant recruitment began after the Institutional Review Board of National Yang Ming Chiao Tung University (Taiwan) approved this study (YM107120F). Additionally, the ethical standards listed in the Declaration of Helsinki were followed throughout the study. The participants could withdraw from the study at any time during the study period and individuals were assured that any potentially identifiable images or data included would not be included in this article. A cooperative research plan for the study was explained to the participants and family members, and both of these groups provided written informed consent that was approved by the Ministry of Legal Consent, and written informed consent was obtained from the participants. No particular ethical issues arose concerning the study design or conduct. Codes were used to represent the names of the participants to ensure privacy and confidentiality.

## Results

### Participants’ Demographic and Clinical Characteristics

The flow chart of participant selection is shown in Figure 1. Thirty-five participants completed the baseline assessment, and 29 and 22 participants completed the 5th and 9th week assessments, respectively. The chi-square test was used to analyze the participants’ demographic characteristics (Table 1). No statistically significant differences were observed in the demographic characteristics (educational level, marital status, sleep pattern, dementia type, dementia severity, and source at baseline or in the 5th and 9th weeks) between the experimental and comparison groups. This finding indicated a homogeneous distribution of participants. Conversely, a statistically significant difference was observed in sex, with male participants comprising 5.9 and 33% of the experimental group and comparison group, respectively (*P* < 0.05); most of the participants were female. The Mann–Whitney *U* test was used to assess the baseline age, NPI and MMSE scores, and medication, and the results showed no significant differences in age, medication, and MMSE scores. However, a statistically significant difference (NPI mean of 36 fractions for the experimental group and 21 fractions for the comparison group; *P* < 0.006) was observed in the NPI scores between the two groups. The experimental group had more serious BPSD than the comparison group at baseline.

**TABLE 1:** Between-group comparisons of the demographic data at baseline, the 5th week, and the 9th week.

Characteristic	Time 1	Experiment (*n* = 17)		Control (*n* = 18)		Chi-square test		Time 2	Experiment (*n* = 13)		Control (*n* = 16)		Chi-square test		Time 3	Experiment (*n* = 11)		Control (*n* = 11)		Chi-square test	
												
		*n*	(%)	*n*	(%)	χ^2^	*P*		*n*	(%)	*n*	(%)	χ^2^	*P*		*n*	(%)	*n*	(%)	χ^2^	*P*
Educational level	Week 0					6.17	0.18						3.99	0.40	Week 9					2.5	0.47
1. Literate		6	(35.3)	2	(11.1)				4	(30.8)	2	(12.5)				4	(36.3)	1	(9.1)		
2. Elementary school (≤6 years)		6	(35.3)	6	(33.3)				4	(30.8)	5	(31.3)				3	(27.3)	5	(45.4)		
3. Junior high school (7–9 years)		3	(17.6)	2	(11.1)				3	(23.1)	2	(12.5)				2	(18.2)	2	(18.2)		
4. 4. Senior high school or college (10–12 years)		2	(11.8)	6	(33.3)				2	(15.4)	5	(31.2)				2	(18.2)	3	(27.3)		
5. University or higher (≥ 13 years)		0	(0.0)	2	(11.1)				0	(0.0)	2	(12.5)				0	(0.0)	0	(0.0)		
Sex						4.11	0.04*						4.90	0.02*						3.47	0.06
1. Male		1	(5.9)	6	(33.3)				0	(0.0)	5	(31.3)				0	(0.0)	3	(27.3)		
2. Female		16	(94.1)	12	(68.7)				13	(100.0)	11	(68.8)				11	(100.0)	8	(72.7)		
Marital status						3.3	0.18						4	0.13						1.2	0.54
1. Unmarried		2	(11.8)	0	(0.0)				2	(15.4)	0	(0.0)				1	(9.1)	0	(0.0)		
2. Married		4	(23.5)	8	(44.4)				3	(23.1)	8	(50.0)				2	(18.2)	3	(27.3)		
3. Widowed		11	(64.7)	10	(55.6)				8	61.5)	8	(50.0)				8	(72.7)	8	(72.7)		
Sleep pattern						3.81	0.14						4.17	0.12						5.13	0.07
1. Advanced sleep-wake phase disorder		0	(0.0)	0	(0.0)			Week 5	0	(0.0)	0	(0.0)				0	(0.0)	0	(0.0)		
2. Delayed sleep-wake phase disorder		6	(35.2)	2	(11.1)				5	(38.5)	2	(12.5)				5	(45.4)	1	(9.1)		
3. Sleep-wake rhythm		8	(47.1)	14	(77.8)				5	(38.5)	12	(75.0)				3	(27.3)	8	(72.7)		
4. Irregular sleep-wake rhythm disorder		3	(17.7)	2	(11.1)				3	(23.0)	2	(12.5)				3	(27.3)	2	(18.2)		
Dementia type						7.64	0.054		11	(84.6)	9	(56.2)	5.61	0.13		9	(81.8)	6	(54.5)	4.40	0.22
1. Alzheimer’s dementia		15	(88.2)	10	(55.6)				0	(0.0)	2	(12.5)				0	(0.0)	1	(9.1)		
2. Parkinson’s dementia		0	(0.0)	3	(16.7)				1	(7.7)	5	(31.3)				1	(9.1)	4	(36.4)		
3. Vascular dementia		1	(5.9)	5	(27.8)				1	(7.7)	0	(0.0)				1	(9.1)	0	(0.0)		
4. Frontotemporal dementia		1	(5.9)	0	(0.0)																
Severity of dementia						1.93	0.38		2	(15.4)	5	(31.2)	1.93	0.38						1.43	0.48
1. Mild		3	(17.7)	7	(38.9)				7	(53.8)	7	(43.8)				1	(9.1)	3	(27.3)	1.89	0.38
2. Moderate		9	(52.9)	7	(38.9)				4	(30.8)	4	(25.0)				8	(72.7)	5	(45.4)		
3. Severe		5	(29.4)	4	(22.2)											2	(18.2)	3	(27.3)		
Source						0.25	0.61						0.16	0.68						0.21	0.64
1. Community		11	(64.7)	13	(72.2)				8	(61.5)	11	(68.7)				8	(72.7)	7	(63.6)		
2. Nursing home		6	(35.3)	5	(27.8)				5	(38.5)	5	(31.3)				3	(27.3)	4	(36.4)		

PS: *P < 0.05.

*All indexes are presented as the number of cases (n) and percentage (%).*

*Thirty-five participants completed the baseline assessment, and 29 and 22 participants completed the 5th and 9th week assessments, respectively. The chi-square test was used to analyze the participants’ demographic characteristics.*

*No statistically significant differences were observed in the demographic characteristics (educational level, marital status, sleep pattern, dementia type, dementia severity, and source at baseline or in the 5th and 9th weeks) between the experimental and comparison groups.*

*This finding indicated that a homogeneous distribution of participants.*

*Conversely, a statistically significant difference in sex was observed, with male participants comprising 5.9 and 33% of the experimental group and comparison group, respectively (P < 0.05).*

### Primary Outcomes

#### Effect of Bright Light Therapy on Behavioral and Psychological Symptoms of Dementia

**FIGURE 3:**
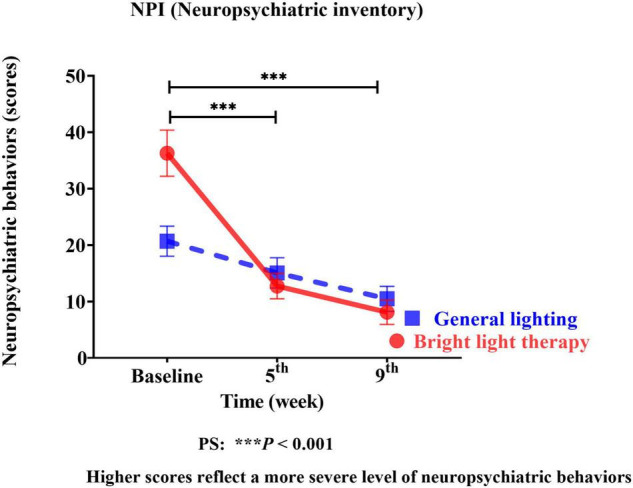
The experimental group showed significant improvement in the NPI (Wald’s test = 12.59, *P* < 0.001; Wald’s test = 10.39, *P* = 0.001) from baseline to the 5th and 9th weeks compared with the comparison group.

We used the NPI as the neuropsychiatric behavior outcome indicator of the effects of bright light intervention. The experimental group showed a significant improvement in the NPI (Wald’s test = 12.59, *P* < 0.001; Wald’s test = 10.39, *P* = 0.001) from baseline to the 5th and 9th weeks compared with the comparison group. The main effect on BPSD was evidenced by a significant change in the slope, reflecting improvements in BPSD (Figure 3). The experimental group showed a significant improvement in the NPI, with a mean decrease of 65% (calculation method: pretest-posttest/pretest) and 78% in the 5th and 9th weeks, respectively. However, the differences in the NPI at the 5th and 9th weeks were not significant (Wald’s test = 0.2, *P* = 0.65).

#### Effect of Bright Light Therapy on Cognitive Function

**FIGURE 4:**
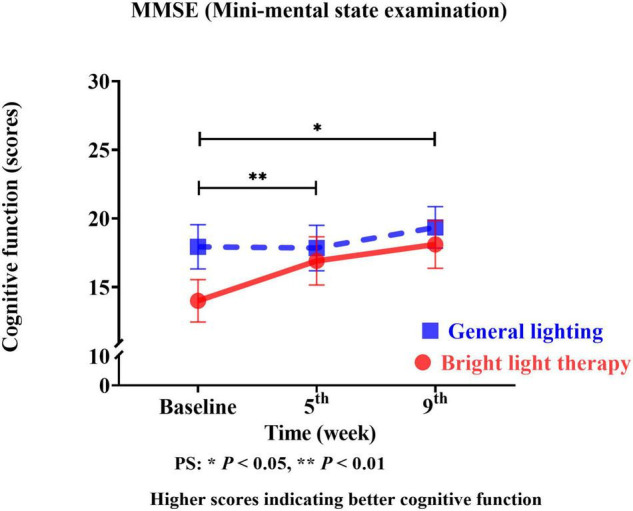
The experimental group showed a significant improvement in the MMSE score (Wald’s test = 7.2, *P* < 0.007; Wald’s test = 3.9, *P* = 0.04) from baseline to the 5th and 9th weeks compared with the comparison group.

We used the MMSE as the cognitive function outcome indicator of the effects of the group bright light therapy intervention. The experimental group showed significant improvement in the MMSE score (Wald’s test = 7.2, *P* < 0.007; Wald’s test = 3.9, *P* = 0.04) from baseline to the 5th and 9th weeks compared with the comparison group. A main effect on cognitive function was observed. A significant change in the slope reflects improved cognitive function (Figure 4). The experimental group showed a significant improvement in the MMSE score, with mean increases of 19% (calculation method: posttest-pretest/pretest) and 28% in the 5th and 9th weeks, respectively. However, the MMSE scores in the 5th and 9th weeks were not significantly different (Wald’s test = 1.5; *P* = 0.20).

### Secondary Outcomes

**FIGURE 5:**
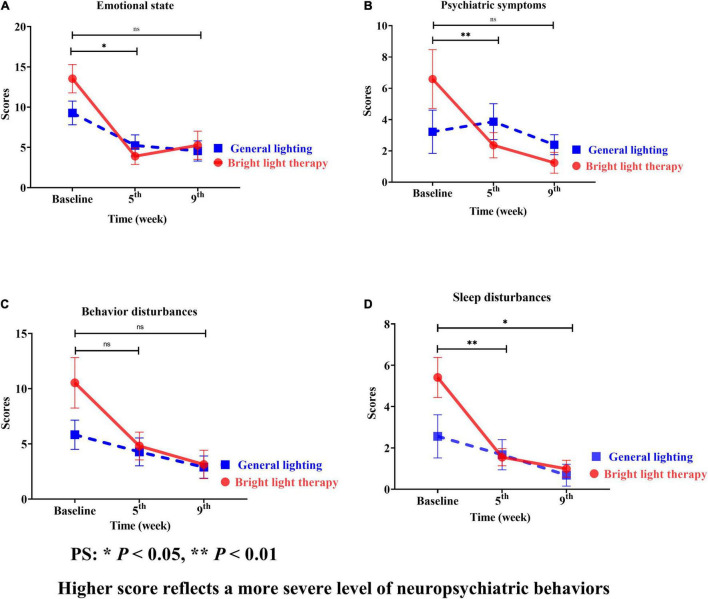
**(A)** Emotional state, **(B)** psychiatric symptoms, **(C)** behavioral disturbances, and **(D)** sleep disturbances of the two groups at different time points; for both the experimental and comparison groups, the domain with the maximum change was psychiatric symptoms. However, the change was not statistically significant **(B)**. Continuous promising improvements were observed in sleep disturbances **(D)**. The experimental group showed a significant improvement in sleep disturbances (Wald’s test = 3.9, *P* < 0.002; Wald’s test = 10.0, *P* = 0.04) from baseline to the 5th and 9th weeks compared with the comparison group.

The NPI is categorized into assessments of the emotional state, psychiatric symptoms, behavioral disturbances, and sleep disturbances (Figures 5A–D). Regarding the specific neuropsychiatric behavioral domains of the emotional state, psychiatric symptoms, and sleep disturbances, continuous, promising improvements were observed in sleep disturbances (Figure 5D). The experimental group showed significant improvement in sleep disturbances (Wald’s test = 3.9, *P* < 0.002; Wald’s test = 10.0, *P* = 0.04) from baseline to the 5th and 9th weeks, which was greater than that in the comparison group.

The experimental group showed a greater mean improvement than the baseline; the highest value was observed for sleep disturbances, followed by psychiatric symptoms, emotional state, and behavioral disturbances. However, the greatest mean improvement in the comparison group was observed for sleep disturbances, followed by emotional state, behavioral disturbances, and psychiatric symptoms. For both the experimental and comparison groups, the domain with the maximum change was psychiatric symptoms (Figure 5B).

**FIGURE 6:**
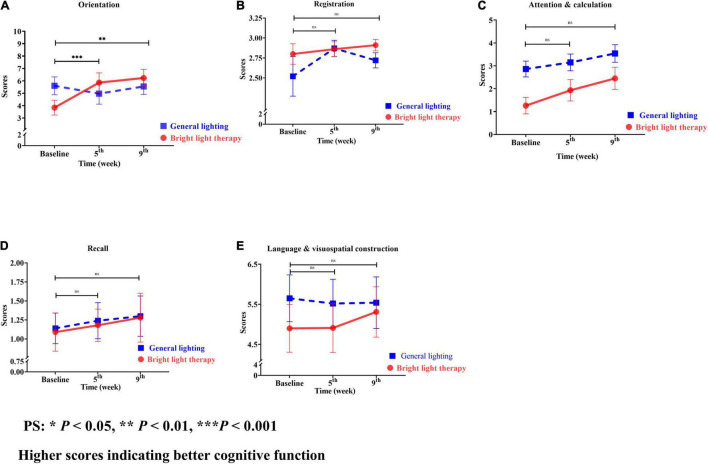
**(A)** Orientation, **(B)** registration, **(C)** attention and calculation, **(D)** recall, and **(E)** language and visuospatial construction of the two groups at different time points. For both the experimental and comparison groups, the domain with the maximum change was orientation. The experimental group showed a significant improvement in orientation (Wald’s test = 10.1, *P* = 0.001; Wald’s test = 8.73, *P* = 0.003) from baseline to the 5th and 9th weeks compared with the comparison group **(A)**.

The MMSE is categorized into orientation, registration, attention and calculation, recall, and language visuospatial construction (Figures 6A–E). Regarding the specific cognitive function domains, a significant change in the slope reflected a better status in terms of orientation, registration, attention calculation, recall, and language visuospatial construction. Among these domains, orientation showed the greatest improvement (Figure 6A). The experimental group showed a significant improvement in orientation (Wald’s test = 10.1, *P* = 0.001; Wald’s test = 8.73, *P* = 0.003) from baseline to the 5th and 9th weeks, which was greater than that of the comparison group.

The experimental group showed greater improvements in attention and calculation, followed by orientation, recall, language visuospatial construction, and registration compared with the baseline value. However, the greatest improvements in the comparison group were observed for attention and calculation, followed by recall, registration, language visuospatial construction, and orientation. For both the experimental and comparison groups, the domain with the maximum change was orientation (Figure 6A).

## Discussion

In this pilot study, bright light therapy was more effective than general lighting at improving BPSD and cognitive function among older adults with dementia. Bright light therapy reduced BPSD and enhanced cognitive function. Although the outcomes at the 5th and 9th weeks were not significantly different, 4 weeks of bright light therapy achieved a significant effect. Therefore, 4 weeks of therapy is recommended because it also requires a comparatively shorter duration of high adherence and acceptability from the participants. Regarding the NPI subdomains, a significant improvement in sleep disturbances was observed. The NPI subdomain with the greatest mean improvement was sleep disturbances for both the experimental and comparison groups; the domain with the maximum change for both groups was psychiatric symptoms. Regarding specific cognitive domains, the domains with the greatest mean improvement were attention and calculation for both the experimental and comparison groups; the domain with the maximum change in the two groups was orientation. Based on previous studies, light exposure suppresses melatonin secretion, decreases 5-HT transporter binding and induces serotonin secretion, mitigates geriatric depressive and anxious moods and improves attentiveness. Additionally, daylight reduces phase delay and night awakening, reducing sleep disturbances (Shikder et al., 2012; Forbes et al., 2014; Missotten et al., 2019; Wirz-Justice and Benedetti, 2020). We identified a light exposure experimental group and comparison group with improvements in attention, calculation, and sleep disturbances. However, the improvement in the experimental group was greater.

### The Bright Light Intervention Model Increased Comfort and Safety and Improved Participant Adherence

These results were consistent with those reported by Fernandez et al. (2018). The authors found that light is effective at modulating sleep and circadian rhythms *via* an indirect pathway and directly affects mood without affecting the sleep pathway or circadian rhythmicity (Rosenberg et al., 2012). We found that bright light therapy is effective at modulating sleep *via* an indirect pathway or even by directly affecting psychiatric symptoms. Our results for general cognition are consistent with those reported in the literature (Yamadera et al., 2000; Riemersma-van der Lek et al., 2008; Chiu et al., 2017; Mitolo et al., 2018; Missotten et al., 2019; Rubiño et al., 2020). However, the results for BPSD are inconsistent with those presented in the literature (Dowling et al., 2007; Riemersma-van der Lek et al., 2008). The difference may be attributed to the use of ambient light that is more friendly to participants than a lightbox (Dowling et al., 2007; Riemersma-van der Lek et al., 2008), which restricts freedom of movement. The differences in duration and dosages, namely, 2,500 lux in the present study and 1,000 lux in the literature (Riemersma-van der Lek et al., 2008), may also explain the discrepancy. Based on previous studies, we assumed that the factors listed below were important when measuring the effects of light therapy on BPSD and cognitive function. (1) Bright light intensity with horizontal illumination of 2,500 lux (van Maanen et al., 2016; Chiu et al., 2017; Missotten et al., 2019) and within a 45° visual field (McCurry et al., 2005, 2011). Current meta-analytic evidence suggests that light therapy is effective at a threshold of ≥ 2,000 lux light intensity (Chiu et al., 2017). Thus, we used bright light (horizontal illumination of 2,500 lux within a 45° visual field). This angle allows bright light to enter evenly and effectively without causing discomfort to the participants. (2) At least 60 min of exposure/day from 9 am to 10 am (Skjerve et al., 2004; Burgess and Emens, 2016; Missotten et al., 2019). (3) The use of group-based ambient light therapy to promote acceptability and patient adherence to treatment (van der Wardt et al., 2017; Vseteckova et al., 2018). Bright light exerted a significant effect, suggesting that 4 weeks is the most effective protocol. This result was consistent with previous findings revealing that light therapy for at least 4 weeks was effective (Figueiro et al., 2014; Figueiro, 2017). In the literature, the lack of illumination for the most effective light exposure intervention duration did not produce consistent conclusions. In the present study, 4 weeks of bright ambient light exerted a significant effect. Light exposure interventions require that treatment acceptability and patient treatment adherence be considered (van der Wardt et al., 2017). Ambient light therapy is achieved by installing high-intensity and low-glare overhead lighting. This approach relies on passive exposure with consistently maintained effectiveness parameters, permitting persons with dementia to engage in their normal activities without restricting their movement, and removing the interaction bias inherent in lightbox studies in this population. The effectiveness of light exposure interventions strongly depends on the patient’s adherence throughout the treatment (van der Wardt et al., 2017; Vseteckova et al., 2018).

### Circadian Rhythms and Sleep

Sleep is fragmented and disturbed in older adults with dementia. Their sleep problems vary according to dementia subtypes, such as Alzheimer’s disease, Parkinson’s disease, and dementia with Lewy bodies. While various types of dementia have been identified, Alzheimer’s disease (AD) is the most common form (Peter-Derex et al., 2015; Sekiguchi et al., 2017). Sleep disruption is frequently observed in patients with dementia and neurodegenerative disease. Sleep disruption alters sleep-wake timing, destabilizes physiology, and promotes a range of pathologies (e.g., neuropsychiatric diseases, metabolic diseases, neuroinflammation, and immunomodulatory diseases) that are considered associated with abnormal sleep. Circadian rhythms are disordered in elderly individuals; they affect not only sleep but also the endocrine system, leading to diabetes, hypertension, metabolic diseases, cardiovascular disease, brain function, cognition, and a decrease in immunity (Wulff et al., 2010; Salami et al., 2011; Urrestarazu and Iriarte, 2016; Cardinali and Brown, 2020; Wirz-Justice and Benedetti, 2020). These effects of light mediated by ipRGCs involve pacemaker-independent input to the suprachiasmatic nucleus (SCN) (Fernandez et al., 2018; Yanar and Halassa, 2018). The SCN is vulnerable to the aging process, resulting in a decreased volume and decreased cell number sensitivity to input signals such as light. Bright light is the first-line treatment to induce circadian resynchronization and ameliorate sleep disturbances. Bright therapy with morning light is usually superior to evening light (Touitou and Haus, 2000; Chang et al., 2018; Rubiño et al., 2020).

### The Feasibility of the Lighting Intervention Model

The attendance rate of the participants was 84%. The feasibility analysis showed that the participants in the experimental group were highly engaged during the intervention. The difference may result from the willingness of the participants’ family members to accompany them to the therapy and provide other assistance, as well as our use of social networking tools—namely, Line—to send participants entertaining and interesting videos and to convey to family members the importance of their participation, particularly in accompanying the participants to the therapy. Family members feedback from the participation process throughout the intervention of bright light therapy also improved their sleep quality and established good interpersonal interactions in their homes. Future research on bright light therapy may improve the sleep quality of people with dementia and their caregivers. A retention rate of 64.7% for participants in the experimental group and 61.1% for the comparison group was observed, and no adverse events were reported. Therefore, the distribution of the participants was homogeneous. Conversely, a statistically significant difference in the sex distribution was found. Due to the sex differences, male participants disliked group activities and withdrew at a higher rate than female participants. Future experimental interventions must consider males’ preferences.

### Limitations of the Study

This study has several limitations. First, this controlled pilot study is based on a convenience sampling design. This procedure might have affected the generalizability of the results. However, the process of compromising because of the practical limitations of people with dementia makes this batch of data more valuable. A randomized controlled trial should be conducted with this intervention in the future. Second, the experimental group showed a greater attendance rate than the comparison group, a finding that may have also affected the results. Third, the comparison group included more male participants than the experimental group, and the sex ratio of the two groups was significantly different. Thus, we were unable to determine the effects of light exposure on BPSD and cognitive function in older male adults with dementia. Other limitations of this study include the patients’ dementia severity and controlled exposure to natural bright light.

### Future Directions

A longitudinal experimental research design is important. Therefore, future research is needed to develop intelligent lighting with variable doses and durations for treating patients with different NPI and MMSE scores by determining the accurate illuminance dose through physiological feedback. We recommend the use of the Clinical Dementia Rating to assess dementia severity in future studies. We suggest that bright light therapy may cause different results for patients with different types of dementia, i.e., Parkinson’s dementia, Alzheimer’s disease, and frontotemporal dementia. These different types of subjects might be included in future studies.

## Conclusion

In this pilot study, we found that the bright light therapy model reduced BPSD and enhanced cognitive function. Improvements in the subdomains of sleep disturbances and orientation function are the key to postponing the deterioration associated with dementia. A 4-week bright ambient light intervention was the most effective.

This intervention, which is a feasible and practical treatment model for older adults with dementia, can be easily replicated. Environmental light exposure interventions may be commonly used in hospitals, nursing homes, daycare centers, community service bases, and even at home.

## Data Availability Statement

The original contributions presented in the study are included in the article/supplementary material, further inquiries can be directed to the corresponding author.

## Ethics Statement

The studies involving human participants were reviewed and approved by the Institutional Review Board of National Yang Ming Chiao Tung University (protocol no: YM107120F). The patients/participants provided their written informed consent to participate in this study.

## Author Contributions

All authors conceived and contributed to designing the experiments and wrote the manuscript and approved it in its final form.

## Conflict of Interest

The authors declare that the research was conducted in the absence of any commercial or financial relationships that could be construed as a potential conflict of interest.

## Publisher’s Note

All claims expressed in this article are solely those of the authors and do not necessarily represent those of their affiliated organizations, or those of the publisher, the editors and the reviewers. Any product that may be evaluated in this article, or claim that may be made by its manufacturer, is not guaranteed or endorsed by the publisher.

## Footnotes

1
http://xds.ym.edu.tw/sl
